# Laparoscopic transabdominal preperitoneal (TAPP) groin hernia repair using n-butyl-2-cyanoacrylate (LiquiBandFIX8) for mesh fixation and peritoneal closure: case report about extraperitoneal hematoma

**DOI:** 10.1097/MS9.0000000000001439

**Published:** 2023-11-07

**Authors:** Sandra Raab, Livia Huber, René Fortelny, Andreas Shamiyeh

**Affiliations:** a Department of General and Visceral Surgery, Kepler University Hospital; b Johannes Kepler University, Linz; c Department for Urology, Hospital Baden-Mödling, Baden, Austria; d Private Clinic Confraternity, General, and Visceral Surgery, Vienna, Austria

**Keywords:** case report, hematoma, inguinal hernia, LiquiBandFIX8, mesh fixation, peritoneal closure, postoperative bleeding

## Abstract

**Introduction:**

A symptomatic inguinal hernia is a prevalent condition that typically requires surgical intervention. Various surgical approaches have been established for hernia repair, including several techniques for peritoneal closure and mesh fixation in laparoscopic surgery. N-butyl-2-cyanoacrylate, such as LiquiBandFIX8, offers a time-saving alternative to invasive methods for both mesh fixation and peritoneal closure. While n-butyl-2-cyanoacrylate is employed in various closure procedures, LiquiBandFIX8 is specifically designed for mesh fixation in inguinal hernia repair.

**Case presentation:**

We present a case of a 68-year old man undergoing transabdominal preperitoneal inguinal hernia repair under full heparinization. LiquiBandFIX8 was employed for mesh fixation and peritoneal closure. Upon conducting a revision laparoscopy due to a significant postoperative hematoma, we found that the mesh and peritoneum remained undamaged and fully sealed, indicating an effective fixing technique. Both the initial repair and the subsequent revision surgery were documented and the videos were subsequently analyzed.

**Conclusion:**

LiquiBandFIX8 provides a reliable adhesive strength and appropriate application for peritoneal closure and mesh fixation. When encountering extraperitoneal fluid collection, there is no anticipation of intraabdominal complications.

## Introduction

Highlights

- Cyanoacrylate glue is an easy tool for peritoneal closure, which does not require high expertise.
- LiquiBandFIX8 can be used for mesh fixation and peritoneal closure.
- A closed peritoneum after laparoscopic transabdominal preperitoneal inguinal hernia repair can withstand high pressure.

With a lifetime prevalence ranging from 27 to 43% in males and 3 to 6% in females, groin hernias and their surgical repair are among the most common medical conditions, with over 20 million procedures performed globally^1^. Virtually all hernias cause symptoms and surgery is the only definitive remedy^2^. The 2018 guidelines recommend mesh fixation as the primary choice during laparoscopic transperitoneal procedures^3^. Using fibrin sealant for mesh fixation has been associated with a reduction in postoperative complications and a shorter procedure duration compared to staples^4^. Subsequently, the peritoneum is typically closed using running sutures^5^.

The aim of this case report is to demonstrate that the adhesive technique for mesh fixation and peritoneal closure using n-butyl-cyanoacrylate (LiquiBandFIX8, Advanced Medical Solutions Limited, Western Wood Way, Langage Science Park, Plymouth, Devon PL7 5BG, UK) can also be safely employed as an adequate alternative expanding its application beyond sutures or tacks. To our knowledge there is no other trial reporting results of adhesive strength following hematoma in the surgical field. This case has been reported in line with the SCARE (Surgical CAse REport) criteria^6^.

## Case presentation

**Figure 1:**
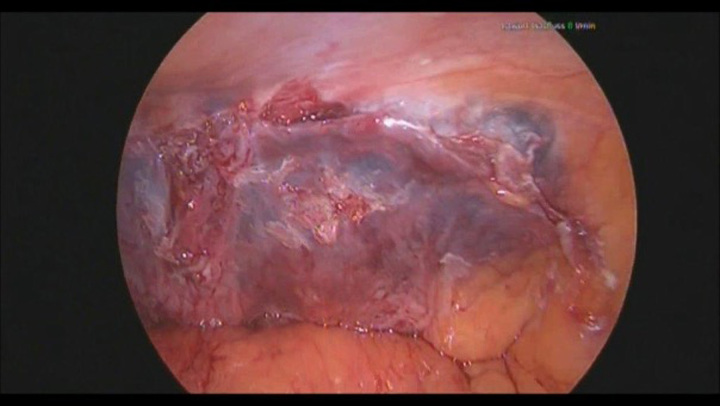
Primary peritoneal closure with LiquiBandFIX8.

Our male patient (68 years) with symptomatic inguinal hernia underwent bilateral transabdominal preperitoneal inguinal hernia repair (TAPP), full heparinized due to aortic replacement and heart valve. Mesh fixation and peritoneal closure were done using n-butyl-cyanoacrylate. The bilateral TAPP procedure was performed by an experienced laparoscopic surgeon. By using a TiO_2_ Bilateral Mesh 30×11 cm (Advanced Medical Solutions Limited) fixated with n-butyl-2-cyanoacrylate (LiquiBandFIX8), a bilateral correction of the hernias was accomplished. The mesh was punctually fixated with LiquiBandFIX8. Subsequently, it was also used for closing the peritoneum (Fig. 1). Beside all three trocar sites, they were injected with local anesthetics.

**Figure 2:**
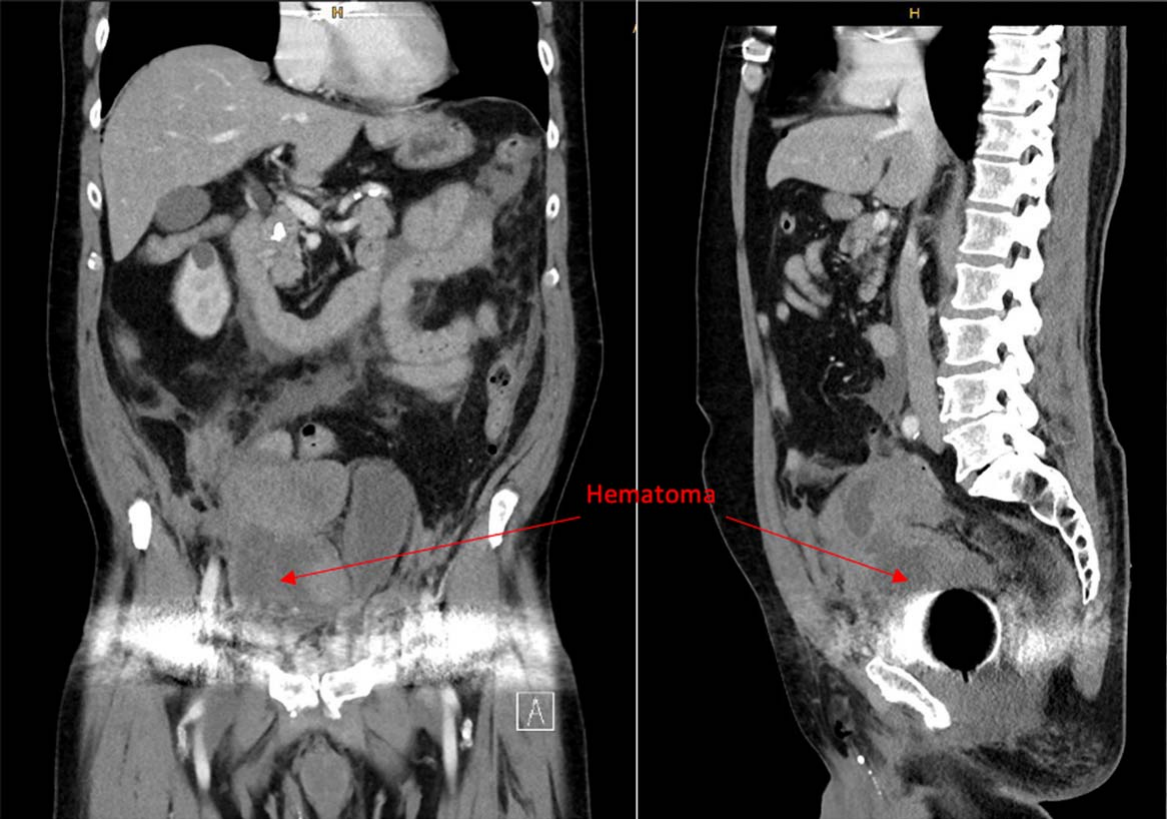
Computed tomography scan with supra-symphyseal hematoma left (arrow: hematoma).

To ensure quality control, all surgeries performed at the Kepler University Surgical Department are meticulously recorded, and critical stages of the procedure are documented through photographs. Two days after the surgery, the patient presented with supra-symphyseal swelling, along with pain and a drop in hemoglobin levels. A computed tomography (CT) scan subsequently confirmed the presence of a sizable hematoma measuring 8×12×13 cm, extending up to the umbilicus, as depicted in Figure 2.

**Figure 3:**
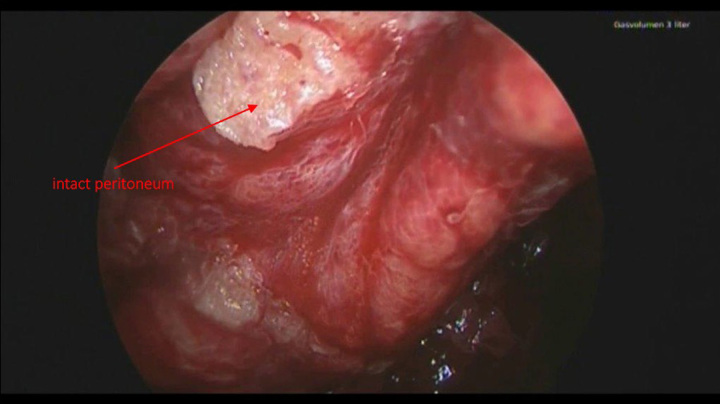
Peritoneum at laparoscopic revision with sufficient glue sealant above retroperitoneal hematoma.

Therefore, we opted to conduct a laparoscopic revision, focusing on evacuating the hematoma and achieving hemostasis. During this revision laparoscopy, it was remarkably evident that, despite containing an extraperitoneal hematoma measuring 700 ml, the peritoneum maintained its integrity and withstood the substantial pressure, as depicted in Figure 3. Upon reopening the peritoneum and removing the hematoma, the mesh remained unharmed and perfectly positioned. Although a specific bleeding source was not identified, it was attributed to diffuse bleeding stemming from full heparinization. Subsequently, the patient experienced an uneventful recovery and was discharged with bilateral groin hernia repair after 5 days. During a follow-up clinical examination one year later, the patient remained entirely asymptomatic, with no indications of hernia recurrence.

## Discussion

In summary, the peritoneal closure resisted the pressure of the 8×12×13 cm postoperative hematoma and was undamaged. No specific bleeding source could be identified. Therefore, it is highly probable that the bleeding was diffuse and attributed to full heparinization. The revisional surgery was filmed and affirmed the integrity of the glue seam despite the pressure of the hematoma. As a 5 mm nonpenetrating laparoscopic fixation tool, it is scheduled as an internal application of cyanoacrylate adhesive (0.0125 g per trigger =33 triggers) during laparoscopic procedures for attaching hernia meshes^7^. The recommended approach involved exclusively applying the adhesive around the defect and along the mesh’s edges. N-butyl-2-cyanoacrylate is the preferred substance for mesh fixation in view of stability and adhesive strength compared to fibrin glue, tolerating a higher abdominal pressure^8^. N-butyl-2-cyanoacrylate is employed in various closure techniques including cutaneous closure, managing bleeding, or addressing corneal perforations^9–11^. Peritoneal closure, in particular, can be accomplished easily using LiquiBandFIX8^7,12^. The treatment procedure of inguinal hernias can be done openly (e.g. Lichtenstein), laparoscopically [TAPP, TEP (laparoscopic totally extraperitoneal inguinal hernia repair)], with penetrating fixation of the mesh (e.g. staples), no penetrating fixation procedure (glue), or with no fixation at all^5,13^. Meshes without fixation do not sufficiently endure pressure^8^. Regarding the recurrence of groin hernias, a great meta-analysis (*n*=3966) showed no differences between open and laparoscopic surgery. Likewise, the recurrence rate is similar in terms of mesh fixation^14^. Despite that, laparoscopic repair was associated with a reduced rate of acute pain compared to open repair and also reduced odds of chronic pain^15^. The current international guidelines for groin hernia treatment recommend a mesh fixation in both surgical methods as the standard of care^3^. Postoperative chronic pain appears within 5–20% of the patients and sexual dysfunction within 9.4% as a negative effect on a patient’s life^16–18^. The application of fibrin glue for mesh fixation reduces the risk of postoperative chronic pain^14^. The primary complications, both in the early and late stages, include hematoma, wound infections, damage to the ductus deferens or nervus ilioinguinalis, and recurrence of the hernia. The application of a mesh combined with fibrin sealant fixation has been shown to mitigate these risks^3,19–21^. In a study conducted by Kukleta *et al*.^22^ in 2012 involving 1300 TAPP operations, it was observed that the utilization of n-butyl-2-cyanoacrylate and other fixation products resulted in perfect mesh integration and the absence of any infections.

## Conclusion

This case report highlights LiquiBandFIX8 as an appealing and secure alternative for fixing the prosthetic mesh and closing the peritoneal defect during laparoscopic hernia repair. Additionally, this procedure is straightforward and efficient with a reduced risk of chronic pain. When used by professionals, it can withstand high pressure, demonstrating its impressive suitability for safe peritoneal closure and mesh fixation. Nevertheless, further studies are essential to validate the effectiveness of n-butyl-cyanoacrylate for both mesh fixation and peritoneal closure to establish a standardized approach.

## Ethical approval

The case report was approved by JKU Medizinische Fakultät (Linz, Austria).

## Consent

Written informed consent was obtained from the patient for the publication of this case report and accompanying images. A copy of the written consent is available for review by the Editor-in-Chief of this journal on request.

## Sources of funding

No funding was received in the writing of the manuscript and in the decision to submit the manuscript for publication.

## Author contribution

A.S.: idea and editing; L.H.: first design; S.R.: revision/imaging/patient’s approval; R.F.: editing.

## Conflicts of interest disclosure

There are no conflicts of interest.

## Research registration unique identifying number (UIN)

Clinical trials: NCT05952791.

## Guarantor

Andreas Shamiyeh.

## Data availability statement

The datasets used and/or analyzed during the current study are available from the corresponding author on reasonable request.

## Provenance and peer review

Not commissioned, externally peer-reviewed.

## Assistance with the study

None.

## Presentation

None.
